# SARS-CoV-2-Specific Immune Response and the Pathogenesis of COVID-19

**DOI:** 10.3390/ijms23031716

**Published:** 2022-02-02

**Authors:** Evgenii Gusev, Alexey Sarapultsev, Liliya Solomatina, Valeriy Chereshnev

**Affiliations:** 1 Laboratory of Immunology of Inflammation, Institute of Immunology and Physiology, Ural Branch of the Russian Academy of Science, 620049 Ekaterinburg, Russia; a.sarapultsev@gmail.com (A.S.); slv10@list.ru (L.S.); v.chereshnev@iip.uran.ru (V.C.); 2 Russian–Chinese Education and Research Center of System Pathology, South Ural State University, 454080 Chelyabinsk, Russia

**Keywords:** adaptive immunity, autoimmunity, cellular stress, cytokines, interferons, post-COVID-19 syndrome, receptors, SARS-CoV-2, superantigens, systemic inflammation

## Abstract

The review aims to consolidate research findings on the molecular mechanisms and virulence and pathogenicity characteristics of coronavirus disease (COVID-19) causative agent, severe acute respiratory syndrome coronavirus 2 (SARS-CoV-2), and their relevance to four typical stages in the development of acute viral infection. These four stages are invasion; primary blockade of antiviral innate immunity; engagement of the virus’s protection mechanisms against the factors of adaptive immunity; and acute, long-term complications of COVID-19. The invasion stage entails the recognition of the spike protein (S) of SARS-CoV-2 target cell receptors, namely, the main receptor (angiotensin-converting enzyme 2, ACE2), its coreceptors, and potential alternative receptors. The presence of a diverse repertoire of receptors allows SARS-CoV-2 to infect various types of cells, including those not expressing ACE2. During the second stage, the majority of the polyfunctional structural, non-structural, and extra proteins SARS-CoV-2 synthesizes in infected cells are involved in the primary blockage of antiviral innate immunity. A high degree of redundancy and systemic action characterizing these pathogenic factors allows SARS-CoV-2 to overcome antiviral mechanisms at the initial stages of invasion. The third stage includes passive and active protection of the virus from factors of adaptive immunity, overcoming of the barrier function at the focus of inflammation, and generalization of SARS-CoV-2 in the body. The fourth stage is associated with the deployment of variants of acute and long-term complications of COVID-19. SARS-CoV-2’s ability to induce autoimmune and autoinflammatory pathways of tissue invasion and development of both immunosuppressive and hyperergic mechanisms of systemic inflammation is critical at this stage of infection.

## 1. Introduction

The pandemic associated with the novel Betacoronavirus (β-CoVs or Beta-CoVs), the severe acute respiratory syndrome coronavirus 2 (SARS-CoV-2) that caused the outbreak of the coronavirus disease 2019 (COVID-19), has been a major public health challenge worldwide [1].

On 31 December 2019, the WHO China Country Office was informed of cases of pneumonia of unknown etiology detected in Wuhan (Hubei Province of China), which would later be considered the center for the spread of SARS-CoV-2. The current emergence of COVID-19 is already a third severe epidemic caused by β-CoV in humans over the past two decades, after the Severe Acute Respiratory Syndrome (SARS) and the Middle East Respiratory Syndrome (MERS), in 2002 and 2012, respectively [2]. At the same time, SARS-CoV-2, having one of the hardest protective outer shells, is expected to be highly resilient in saliva or other body fluids and outside the body and, thus, possess fecal transmission potential [3].

The pathogenesis of COVID-19 is complex, but it can be conceptually described using typical models for the three main pathological processes associated with inflammation—local manifestations of classical general (canonical) inflammation, acute systemic inflammation, and chronic systemic inflammation of low intensity [4]. The probability of the latter process increases with aging, especially in persons with metabolic syndrome, type 2 diabetes mellitus, and some other severe chronic diseases [4,5].

The other side of the research into COVID-19 pathogenesis is the study of selective virulence and pathogenicity factors that are specific to β-CoV viruses in general, or unique to SARS-CoV-2. These factors determine the specificity of the respective disease. Thus, SARS-CoV-2 employs three distinct sets of conventional viral pathogenetic strategies:

(1) Recognition by the virus by cellular receptors, which can be divided into three functional groups:
  (a) Receptors that enable the virus to penetrate the target cell. To implement this strategy, viruses strive to increase their binding affinity as well as expand the repertoire of these receptors and their coreceptors [6].
  (b) Receptors that transmit to the target cell information useful for the virus (combinations of properties A and B are possible in one receptor).
  (c) Cellular receptors which, after recognizing a virus, initiate an antiviral response. In this case, the virus strategy is to inhibit these receptors and their signaling pathways [6].
(2) Suppression of the antiviral response, from both the infected target cells and the immune system of the host organism. This virus strategy can also be subdivided into several components:
  (a) Inhibition of early antiviral effects of interferons (IFNs) type 1 (INF-I) and type 3 (IFN-III).
  (b) Disruption of universal cellular stress signaling pathways or specific immune pathways.
  (c) Protection of the virus from the direct action of antiviral response factors.
(3) The ability of the virus to provoke immune system aggression against its tissues in the form of an autoimmune and autoinflammatory process is a separate strategy for viral survival in the host body.

New information on the presence of a large number of known and unknown SARS-CoV-2 receptors allows a more realistic assessment of the efficacy of blocking the major viral receptor (ACE2) in COVID-19 therapy. A deeper insight into the phasing and redundancy in the action of the virus’s pathogenicity factors on the immune system drives a need for a more comprehensive approach to COVID-19 pathogenetic therapy.

Thus, the purpose of this review is to systematize the data on the recently revealed mechanisms of virus penetration into target cells, inhibition of antiviral immune defense, and possible pathways for the development of an autoimmune and autoinflammatory process. The review will attempt to integrate and systematize various and internally contradictory mechanisms of SARS-CoV-2 virulence and pathogenicity.

## 2. General Characteristics of SARS-CoV-2 Infection

The SARS-CoV-2 virus belongs to the group of enveloped viruses containing a positive single-stranded RNA genome [7]. It is classified into the order Nidovirales, family Coronaviridae, subfamily Coronavirinae, genus Betacoronavirus [8].

The SARS-CoV-2 genome size is significant and amounts to 29.99 kb [9]. Its organization is similar to that of other CoVs and consists mainly of open reading frames (ORF). About 67% of the SARS-CoV-2 genome is ORF1ab. The latter encodes the synthesis of polyproteins in the infected cell (1a, 1ab). After the synthesis, polyproteins are degraded by two proteases (nsp3 and nsp5) into 16 separate nonstructural proteins (nsp1–16): 11 nsp from the ORF1a segment (nsp1 to nsp11) and 5 from the ORF1b segment (nsp12 to nsp16) [10,11,12]. The remaining 33% of the SARS-CoV-2 genome are represented by the genes of structural and auxiliary (additional) proteins (ORF3a, ORF3b, ORF6, ORF7a, ORF7b, ORF8, ORF9b, ORF10). ORFs are distributed between structural genes and, accordingly, encode viral proteins: orf3a, orf3b, orf6, orf7a, orf7b, orf8, orf9b, orf10 [12,13,14,15,16,17,18,19,20,21]. 

**Figure 1:**
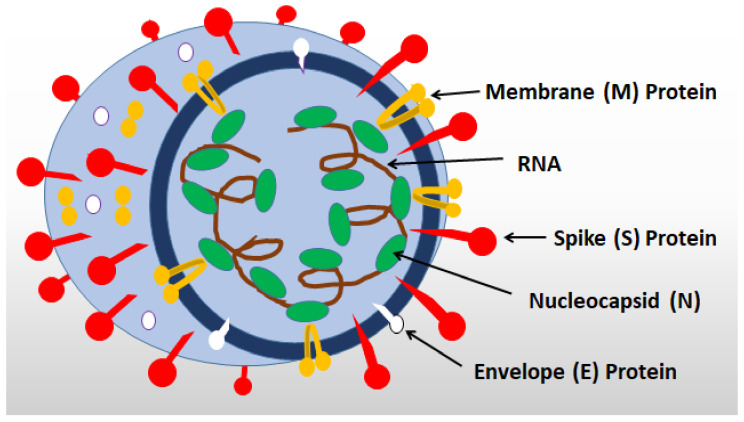
The principal structure of SARS-CoV-2.

The four structural proteins of SARS-CoV-2—(1) spike (S) glycoprotein, (2) small envelope glycoprotein (E), (3) membrane glycoprotein (M), and (4) nucleocapsid protein (N)—are responsible for viral replication and structuring, virus binding with cellular receptors (S), as well as for the pathogenicity of the virus [14,22,23] (Figure 1).

Once assembled, viruses are transported by vesicles to the host cell membrane and released by exocytosis. When being transported to the cell surface, S-protein allows the infected and healthy cells to be fused, resulting in the formation of large multinucleated cells which spread the virus in the host organism [24].

A common feature of all three well-known β-CoVs, namely SARS-CoV-1 (the causative agent of SARS), MERS-CoV, and SARS-CoV-2, is that they can replicate in the lower respiratory tract and cause fatal pneumonia [2]. The likelihood of death increases sharply with the development of acute respiratory distress syndrome (ARDS) [25]. In this case, viral expansion in the body, hypoxia, entry of tissue decay products into the bloodstream, pathological hyperactivation of T cells and macrophages, and intravascular activation of leukocytes, complement systems, and hemostasis lead to a range of resuscitation syndromes pathogenetically associated with systemic inflammation [4,25,26].

The SARS-CoV-2 virus is significantly less lethal than SARS-CoV-1 or MERS-CoV, but it is transmitted much easier and faster [27,28]. The long incubation period and the presence of asymptomatic variants of COVID-19, as well as the high level of contagiousness and transmissibility, make the identification, tracking, and elimination of this disease challenging [27,28].

The main route of infection for SARS-CoV-2 is through respiratory droplets, but contact with an infected surface can be also important [29]. The incubation period of COVID-19 is from three to 14 days and depends on the immune status [30]. The typical symptoms of COVID-19 include fatigue, fever, dry cough, malaise, sore throat, loss of taste and/or smell, and in some cases, shortness of breath, diarrhea, and characteristic signs of viral pneumonia [31].

A special stratum of viruses is the inhibition of the signaling pathways of receptors responsible for triggering antiviral immunity. Primarily, these are pattern recognition receptors (PRRs), which recognize conserved molecular structures known as a pathogen or injury-associated molecular patterns (PAMP and DAMP). PAMPs are associated with microbial pathogens, while DAMPs are associated with the host cell components that are released during cell damage or death. The main PRR families for the recognition of viral RNA in endosomes are Toll-like receptors (TLR), while cytoplasmic viral RNA is recognized by RIG-I-like receptors (RLR) [32]. Activation of these receptors leads to the activation of an antiviral innate immune response, primarily associated with the production of IFNs. Scavenger receptors (SR) are a special group of molecules capable of non-strictly specific interaction with viruses [33]. This is a large group of receptors at the intersection of immunity and metabolism. They are predominantly expressed on stromal macrophages and dendritic cells [34]. Also, SR can act as cofactors of PRRs, including TLRs, in the recognition and neutralization of viruses by cells of innate immunity, but, in some cases, they can act as a gateway for viruses (including SARS-CoV-2) to infect cells [35].

Additionally, a common pattern in the pathogenicity of viruses, including CoVs, is their ability to suppress the production and function of IFNs of type 1 (multiple forms of IFN-α - 13 factors, IFN-β, IFN-ε, IFN-κ, IFN-ω), and type 3 (IFN-λ1–4), triggering hundreds (> 300) of IFN-stimulated genes (ISG) [36,37,38,39,40].

The impact of viruses on the activation mechanisms of cellular stress in immunocompetent cells is also significant, as it causes polyclonal activation and apoptosis of lymphocytes (primarily T cells), pathological activation of macrophages, and immunosuppression [41].

RNA viruses such as CoVs exhibit a much higher evolutionary rate than DNA viruses due to their high susceptibility to replication errors mediated by RNA polymerase or reverse transcriptase, and due to the significant size of the viral population with a higher replication rate [42,43]. Currently, the identification of all SARS-CoV-2 mutations and their connections with pathological changes is almost impossible, mainly because there are asymptomatic patients [24]. However, a global analysis of the known genomic epidemiology of SARS-CoV-2 is available in the public domain [44]. Omicron is characterized by a large number of mutations in the spike protein (more than 30), as well as new mutations in the nsp12 and nsp14 proteins [45,46,47]. Omicron is more transmissible than the Europe-wide spread variant of Delta SARS-CoV-2; it is capable of significant immune evasion (including from the currently used vaccines) and spreads faster than any previous variants of the virus [48,49,50,51,52,53,54].

Overall, the genome of the current epidemic SARS-CoV-2 virus has undergone significant changes compared to the reference genome obtained in January 2020. The most significant mutations that significantly alter virulence and pathogenicity occur in the S-protein [9]. Harmful variations in nsp12, in the N-protein, were also described [55,56]. The spread of new mutations in the S-protein can potentially reduce the effectiveness of the immune response to vaccines [57]. A systematic assessment of all 3686 possible future mutations in the S-protein domain that binds cell receptors shows that future mutations will most likely make SARS-CoV-2 even more infectious [58].

## 3. SARS-CoV-2 Receptors

Coronaviruses bind to host receptors through their spike S-glycoproteins, which mediate membrane fusion and viral penetration [59]. The main receptor for SARS-CoV-2 is membrane angiotensin-converting enzyme 2 (ACE2) [60]. There are two isoforms of ACE2, and one of them cannot bind to SARS-CoV-2 [61,62,63].

**Figure 2:**
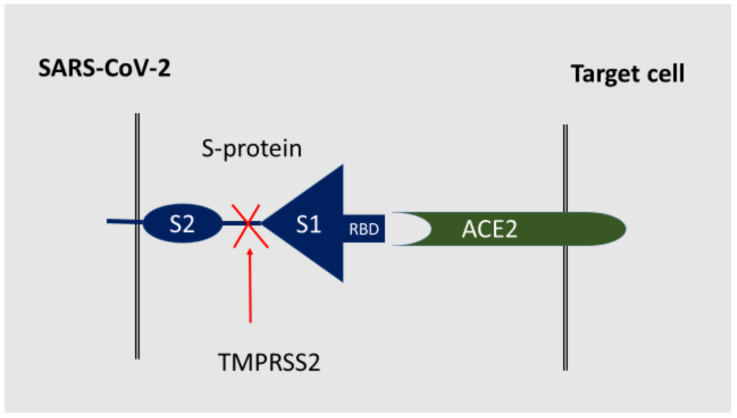
The receptor function of SARS-CoV-2 S-protein. Proprotein convertases (e.g., furin) act after the virus attaches to ACE2. The presence of a furin cleavage site at the S1/S2 border in SARS-CoV-2 probably reduces the dependence on target cell proteases [65]. Cell surface proteases (e.g., TMPRSS2) catalyze the cleavage of S1 and its separation from the S2 domain [64]. Acid lysosomal proteases act after viral endocytosis, in the lysosomal pathway of transformation of the virus in endosomes. Moreover, due to the complexity of in vivo processes and infection of various cells types with SARS-CoV-2, other host proteases can potentially participate in similar cleavage of the SARS-CoV-2 S protein [15].

S-protein forms trimers on the surface of the virus [64]. After RBD-receptor interaction, the S protein undergoes proteolytic cleavage at the N-terminal S1 subunit and the C-terminal S2 subunit of host proteases. This partial proteolysis is catalyzed by the transmembrane protease serine 2 (TMPRSS2) and can be activated by furin or furin-like proteases (e.g., plasmin) or after endocytosis by cathepsins B/L [64]. The receptor-binding domain (RBD) of the S1 subunit directly interacts with the ACE2 (Figure 2). However, in the S-protein trimer, only one of the three RBDs can be turned upward in a receptor-accessible conformation. At the same time, SARS-CoV-2 is in a state of low activity for binding to ACE2 for a considerable time, since RBD is shielded by the carbohydrate component of the S-glycoprotein, which protects the virus from antibodies [65]. Therefore, RBD undergoes a hinge conformational movement that temporarily obscures or exposes receptor-binding determinants [66]. This SARS-CoV-2 property limits the ability to neutralize the virus with antibodies and drugs targeting RBD binding.

Thus, after attachment to the receptor, proteolytic processing activates the S-protein and makes possible the fusion of the membranes of the virus and the target cell, followed by the release of viral RNA into the cytoplasm of the cell. In this case, the distal S1 subunit plays a role in the recognition and binding of the receptor, while the anchored S2 subunit mediates the fusion of the membranes of the virus and the host cell [67]. SARS-CoV-1 also enters cells by endosomal pathways, where the S-protein is activated for the fusion of the viral and endosomal membranes by trypsin-like proteases (e.g., cathepsin B) in an acidic endosomal environment [68]. This path is quite possible for SARS-CoV-2 [69,70]. Many coreceptors acting synergistically with ACE2 can activate the endosomal pathway [65].

Due to differences in the spectrum of ACE2 genetic variants, ACE2 receptors feature different degrees of binding affinity for the S-protein. Several ACE2 variations can form high-affinity double mutant complexes with S-protein, which can influence an individual’s susceptibility to infection [71]. Moreover, hundreds of variants in the RBD domain have been found, of which the mutant type V367F constantly circulating across the world exhibits a greater binding affinity for ACE2 [72]. The presence of numerous mutations in the S-protein also indicates the ability of the spike protein to acquire new properties of ligand specificity [73]. In particular, the importance of searching for alternative ACE2 receptors to SARS-CoV-2 is highlighted by the reports, suggesting that bone marrow cells that do not express ACE2 could be infected with this virus [74].

Recently, several membrane proteins that can act as ACE2 cofactors or alternative receptors have been discovered (Table 1). Thus, S-glycoprotein can interact with receptors not only through its protein part but also by binding to lectin receptors with its carbohydrate component (N-glycans of the S1 subunit, containing oligomannose and complex sugars that protect the virus from antibodies) [15,75,76]. Lectin-like S1 sites, in turn, bind to heparin, which can affect and even prevent viral invasion [77]. On the contrary, binding of lectin-like S1 sites to the target cell glycocalyx can facilitate invasion, since the glycocalyx contains coreceptor sugars for binding SARS-CoV-2, namely, O-acetylated sialic acids [78] and heparan sulfate [79]. It was shown that heparan sulfate could enhance the penetration of many types of viruses [80]. The interaction of the lectin-like S1 domain with the glycocalyx of target cells may have a cofactor significance for the ACE2 receptor function during cell infection with SARS-CoV-2 [79,81]. The possibility of SARS-CoV-2 S protein binding to the integrin receptors of the RGD motif (Arg-Gly-Asp) in the RBD S1 domain is also discussed [82]. At the same time, it is not always clear whether these interactions contribute to viral invasion or virus neutralization.

Many RNA viruses use extracellular vesicles (ectosomes and exosomes) for translocation into new host cells [107,108]. These vesicles allow viruses to infect cells via virus-specific receptors as well as in an independent manner. It was suggested that the cellular transport pathway associated with the release of SARS-CoV-2-loaded extracellular vesicles might represent potential mechanisms for the relapse of COVID-19 infection [109]. In particular, exosomes expressing ACE2, CD9, and other tetraspanins on their surface can be mediators of COVID-19 infection [110]. At the same time, exosomes can transfer viral particles from infected cells to healthy ones and modulate the host’s immune responses, and thus can be exploited for the therapy of COVID-19 [111].

Overall, the entry of SARS-CoV-2 into host cells is a complex, multifactorial process. Even the main mechanism associated with ACE2 requires the involvement of many auxiliary molecules in the process—proteinases, coreceptors, and activators of their expression. The presence of coreceptors, in particular, enables SARS-CoV-2 to infect cells with low ACE2 expression on membranes. Simultaneously, there is increasing evidence of the availability of alternative ACE2 pathways for target cell infection [100]. The variety of mechanisms of SARS-CoV-2 tropism to human tissues can explain its high contagiousness, as well as viral invasion of internal organs during the progression of COVID-19 (Table 1).

However, the preliminary data on the role of CD147 as an ACE2-independent receptor for SARS-CoV-2 [89] were not fully confirmed [90]. The CD147 mechanism appears to be more complex and indirect and associated with the regulation of ACE2 membrane expression [90]. At the same time, it was shown that the scavenger receptor SR-B1, which recognizes high-density lipoproteins (HDL), promotes SARS-CoV-2 penetration in an ACE2-dependent way [35]. The S1 subunit of the virus binds to cholesterol and HDL components, which increases viral uptake. Because SR-B1 interacts with these receptors, SARS-CoV-2 was found to enter cells expressing ACE2 more easily when SR-B1 was expressed. SR-B1 has been reported to co-express with ACE2 in human lung tissue and various extrapulmonary tissues [35]. The fact that SR-B1 has numerous functions, including being a viral scavenger, immunomodulator, and virus penetration intermediate [101], explains its involvement in COVID-19.

The potential ability of the C-type lectin CD209L (L-SIGN) to act as an independent SARS-CoV-1 receptor on target cells was also described [112,113]. In this regard, the attention of researchers was drawn to the report on the identification of CD209L and the related protein CD209 (DC-SIGN) as receptors capable of mediating the penetration of SARS-CoV-2 into human cells [97]. The receptors CD209L and CD209 interact with the ligand-specific (for ACE2) site of the S-protein in RBD. In addition, CD209L also interacts at the cell membrane with ACE2, suggesting a role for CD209L and ACE2 heterodimerization in SARS-CoV-2 penetration and infection in cell types where both are present, such as human endothelial cells. This determines both ACE2-independent and ACE2-dependent roles of CD209L in SARS-CoV-2 entry and infection. CD209L and CD209 were shown to serve as alternative receptors for SARS-CoV-2 in cell types where ACE2 is low or absent. Meanwhile, the capture of CD209 viruses in macrophages and dendritic cells can lead not to the infection of these cells but the utilization of viral particles, whereas in other cases, it leads to the infection of T-lymphocytes in contact with macrophages [99].

Patients with a severe case of COVID-19 demonstrate significant complement activation in the lungs, skin, and serum [114]. Activation of the complement system in COVID-19 occurs in a variety of mechanisms, most of which are linked to the activation of hemostasis systems and kallikrein-kinins, as well as with damage to the vascular endothelium and other tissues [115,116]. SARS-CoV-2, on the other hand, has been demonstrated to activate complement via the lectin pathway [117].

There is evidence of the ability of the SARS-CoV-2 S protein to bind to membrane PRRs, including TLRs, especially the TLR4 [100,118,119]. It is assumed that S-protein binds to TLR4 and activates TLR4 signaling to increase the expression of ACE2 on the cell surface, thereby facilitating the penetration of SARS-CoV-2 into type II alveolocytes [120]. This contributes to cell destruction, disruption of surfactant production, and ARDS development. It is assumed that myocarditis caused by SARS-CoV-2 may be associated with TLR4 activation and subsequent hyperactivation of the innate immune response [120]. The native S-protein SARS-CoV-2 binds to TLR1 and TLR6, though with lower binding energy than TLR4 [119] according to molecular docking studies. It is important to remember that TLRs can form intricate complexes with other receptors in the presence of ligands, including SR, integrins, tetraspanins, and Fc receptors (FcR) [33,34]. This scenario precludes simultaneous recognition of not just unique and characteristic viral antigens but also other alterations in the organism’s genetic and phenotypic homeostasis. These changes, including the accumulation of endogenous PRR ligands, particularly DAMP, in the blood and other tissues, grow like an avalanche as the viral infection progresses.

A single-stranded SARS-CoV-2 RNA can bind TLR7 and TLR8, while double-stranded virus RNA (temporary generated during single-stranded virus RNA replication) can bind to TLR3 in macrophages and dendritic cells [121]. These interactions (through TLRs in endosomes) induce IFN-I responses and the generation of various cytokines, resulting in viral infection suppression [122]. However, PRRs of the RLR family, primarily RIG-I (retinoic acid-inducible gene I) and MDA5 recognize SARS-CoV-2 RNA in the cytoplasm of numerous cell types, inducing IFN-dependent stress in infected cells [37,123]. These receptors interact with viral double-stranded RNA appearing during RNA replication. They have a caspase activation and recruitment domain (CARD) that activates various apoptotic and inflammatory signaling pathways. RLR signaling pathways are associated with antiviral signaling in the mitochondria (mitochondrial antiviral-signaling protein, MAVS). MAVS leads to the production of several multifunctional protein complexes after RLR-dependent activation. On the ER and mitochondrial membranes, RLR-MAVS interacts with STING (stimulator of interferon genes) and TRAF3 (TNFR-associated factor 3), activating TBK1 (TANK-binding kinase 1) and IKK (inhibitor of B kinase epsilon) kinases [114,124]. The transcription factors IRF3 (interferon regulatory factor 3) and NF-κB (nuclear factor-kappa B) are then activated by these kinases [124,125,126]. STING is a critical PRR in this scenario, as it combines responses to viral RNA and DNA, as well as intracellular DAMPs (endogenous DNA trapped in the cytosol) [127,128]. IKK and TBK1 are also involved in the TLR3 (activates TRAF3) and TLR4 (through MyD88) signaling pathways [129,130]. The signaling pathways of RLR, TLR, cytokine receptors, and other signaling structures that induce the production of interferons (especially through the activation of IRF3) and the development of cellular stress in general (especially through the activation of the multifunctional NF-κB) can thus be intertwined during a viral invasion.

In patients with COVID-19, the RLR/MAVS-mediated signaling pathway not only plays a role in the antiviral response, but its failure can also lead to autoimmune disorders and trigger a cytokine storm [37]. Furthermore, SARS-direct CoV-2’s impact on the nod-like-receptor (NLR) and secondary changes in intracellular homeostasis induce viral-nonspecific cellular stress pathways in diverse cells, such as NF-κB activation and NLRP3 inflammasome assembly [131,132]. Inflammasomes, in turn, increase the synthesis of IL-1β and IL-18, as well as pyroptosis, a type of planned necrosis in which a substantial amount of DAMP escapes the cell [133]. Inflammasome development can have both protective and pathogenic implications depending on the circumstances. COVID-19 dysfunction can result in not only harm to host tissues but also the development of systemic inflammation [4]. Other cellular stress pathways that are influenced by SARS-CoV-2 include:

- Oxidative stress [134,135];
- Autophagy and lysosomal stress [136,137];
- Ubiquitination of proteins; this process is important for the regulation of function and proteolysis of many intracellular proteins [138];
- Mitochondrial stress, in particular, is dependent on the RLR/MAVS signaling pathway [139];
- Endoplasmic reticulum (ER) stress [140];
- Expression of non-coding, regulatory RNAs [141,142,143,144];
- Expression of heat shock proteins (HSP) [81,145];
- Cell response to the DNA damage [146,147];
- The formation of a pro-inflammatory cell secretary phenotype, which can manifest itself as a cytokine storm syndrome when generalized to the whole organism.

Thus, the recognition of SARS-CoV-2 target cells and vice versa—the recognition of the pathogen by the sensors of the cell’s antiviral defense is the first stage of virus invasion. In this scenario, it is not only the virus’s main receptor (ACE2) and its coreceptors that are engaged but also the virus’s alternate receptors. This enables SARS-CoV-2 to infect a large range of target cells in a variety of human organs. Several universal and viral invasion-specific cellular stress signaling pathways are triggered during virus penetration into target cells. Furthermore, at the level of individual cells and the organism as a whole, an escalation of the mutual opposition of immune systems, principally IFN-dependent, and the already developed system (virus and recruited proteins) of the virus’s vital factors develop. The outcome of this battle will influence how the infection develops and progresses.

## 4. Pathogenicity Factors of SARS-CoV-2 Overcoming the IFN-Response at the Initial Stages of Viral Invasion

The cell’s response to infection is initiated by viral PAMPs, which are recognized by PRR, followed by activation of downstream signaling molecules such as adapter proteins MAVS and MyD88, kinases TBK1, IKK, transcription factors IRF3, NF-κB, activator protein 1 (AP-1), and others, which results in high IFN-I-III production and pro-inflammatory cellular stress in general. This is referred to as primary IFN production. The result of the PRR’s initial stimulation of cells is a positive feedback loop in the form of the interferon response. IFN-I-III activates signaling pathways involving the kinases Tyk2 (tyrosine kinase 2) and Jak1 (Janus kinase 1), which are directly linked to the intracellular domains of two IFN-I (IFNAR1/IFNAR2) and IFN-III (IL-10R2/IFNLR1) chains. The ISGF3 transcription factor complex (STAT1-STAT2-IRF9) is thus activated and penetrates the cell nucleus, activating the ISG genes [148]. A recent study demonstrated the presence of STAT1-independent mechanisms of ISG promoter activation (through binding to ISRE—interferon-stimulated response element), through the transcription factor complexes STAT2-IRF9 and STAT6-STAT2-IRF9, which are also linked to the function of IFN receptors (IFN-R) [149].

**Figure 3:**
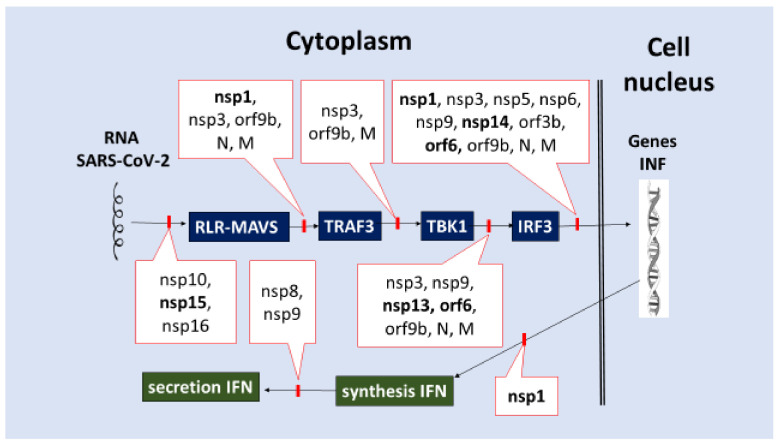
The primary phases of the essential signaling pathway for IFN-I-III production are inhibited by SARS-CoV-2 proteins. The signaling pathway for inducing IFN-I-III production is indicated by the blue color. The green color denotes post-transcriptional steps in IFN-I-III production and secretion. The blocks of the corresponding stages of IFN-I-III induction, production, and secretion that are produced by SARS-CoV-2 proteins are shown in red. The direction of action effects is indicated by arrows.

**Figure 4:**
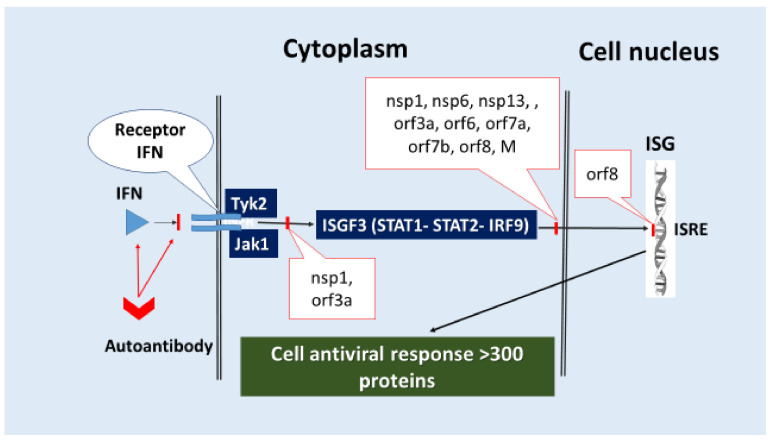
Inhibition of the key IFN-I-III signaling pathways by SARS-CoV-2. Orf8 blocks the attachment of the ISGF3 complex to the ISRE site on the promoters of antiviral response genes—ISG. Additionally, Figure 3 shows the possible inhibitory effect of autoantibodies that bind IFNs. Therefore, the presence of neutralizing autoantibodies to IFN-I is a predictor of critical COVID-19 pneumonia [216,217,218,219]. IFN receptors and signaling routes for activation of ISG genes are colored red, IFN receptors and signaling pathways for activation of ISG genes are colored blue and cyan, and ISG gene products are colored green. The arrows point in the direction of the impacts of the respective molecular structures’ actions.

Furthermore, ISG expression products (OAS, IFITM, and others) protect cells from coronavirus infection by degrading viral RNA and reducing virus penetration [150]. Moreover, IFN-IRs are expressed almost everywhere, but IFN-III-R is only found on cells that line the epithelial barrier [151]. In general, both the production of IFN-I-III and the subsequent transmission of secondary signals by them for the development of antiviral cellular stress are critical for controlling the development of the early stage of coronavirus infection. Overcoming the IFN-dependent processes of innate immunity is a common viral invasion strategy, and COVID-19 is no exception. Inhibition of these processes, in turn, is a prerequisite for viral invasion. The main points of inhibition include:

(1) Inhibition of the formation and function of PRRs that recognize SARS-CoV-2 RNA.
  (A) Non-structural proteins nsp14 (N-7-methyltransferase) and nsp16 (2-Oʹ-methyltransferase) β-CoV can modify viral RNA to prevent it from being recognized by PRR and IFIT1 (interferon-induced protein with tetratricopeptide repeats 1, which inhibits viral replication and translation initiation) [152,153]. The nsp16 factor, in conjunction with nsp10, methylates the viral mRNA’s 5′ end to mimic the host’s mRNA [154,155].
  (B) Factor nsp1 blocks several cell proteins, including antiviral PRRs, from being synthesized on ribosomes, while N and M proteins bind to and inhibit RIG-I. (Table 2).
(2) Inhibition of IRF3 activation and translocation into the cell nucleus or cleavage, which initiates IFN-I-III production. Many of the SARS-CoV-2 proteins (nsp1, nsp3, nsp5, nsp6, nsp9, nsp13, nsp14, nsp15, orf3b, orf6, orf9b, N, and M) can block a variety of signaling pathways, including RLR/MAVS/TRAF3/(IKK and TBK1)/(IRF3, NF-κB)/ (Figure 3).
(3) Inhibition of IFNs synthesis on ribosomes (nsp1, Table 2).
(4) Inhibition of transmembrane transport of IFNs (nsp8, nsp9) (Table 2, Figure 3).
(5) Inhibition of IFNAR1–IFN-IR chain (orf3a), inhibition of Tyk2 kinase associated with IFN-R (nsp1) (Table 2, Figure 4) [156,157,158,159,160,161,162,163,164,165,166,167,168,169,170,171,172,173,174,175,176,177,178,179,180,181,182,183,184,185,186,187,188,189,190,191,192,193,194,195,196,197,198,199,200,201,202,203,204,205,206,207,208,209,210,211,212,213,214,215,216,217,218,219,220].
(6) Direct inhibition of ISG expression by blocking the activation and translocation into the nucleus of the ISGF3 complex, which initiates ISG transcription. For example, nsp1, nsp3, nsp6, nsp13, orf3a, orf6, orf7a, orf7b, orf8, and M (Table 2) have pathogenic capabilities for blocking several signaling pathways, including IFN-I-III/IFN-R (Tyk2, Jak1)/ISGF3 (STAT1, STAT2, IRF9)/ISG/antiviral response, cellular stress (Figure 4).

In general, the pathogenicity factors of SARS-CoV-2 can inhibit all the main stages of IFN protection with a high degree of redundancy due to both the large number of factors involved in this inhibition and their multifunctionality. The synthesis of interferons (IFNs) during viral infection is avalanche-like. As a result, disrupting all developmental phases of the IFN response at the same time is an effective multilateral mechanism that suppresses the host’s antiviral response synergistically [176]. Among the 27–28 viral proteins found in SARS-CoV-2, nsp1 [155] and orf6 [150] were thought to have the greatest impact on both primary IFN-I-III production and signaling to ISG. However, in SARS-CoV-2 infection, nsp13, nsp14, nsp15, and orf6 have also been demonstrated to be powerful interferon antagonists [150]. Thus, it is probable that the repertoire of dominant IFNs inhibitors varies depending on the cell type and state of function. These processes work together to allow SARS-CoV-2 to reproduce in a variety of cell types while also overcoming innate and adaptive immunity’s antiviral capabilities.

Consequently, the complex of SARS-CoV-2 pathogenicity factors aims to prevent IFN-I-III antiviral activity from being formed and implemented [221]. As a result, genetic defects in the generation of IFNs or the presence of autoantibodies that disrupt IFN function are evident risk factors for the severe course of COVID-19 [222]. Suppression of IFN-I-III activity at the outset of the disease is critical for viral invasion, as IFN-I-III has the greatest antiviral effectiveness during this time. Overproduction of IFNs may also play a deleterious effect in the latter and more severe stages of COVID-19, as it is implicated in the pathophysiology of the cytokine storm phenomenon and associated systemic inflammation [4]. As a result, a severe form of COVID-19 with the development of ARDS and an increased level of pro-inflammatory cytokines in plasma, such as IL-1β, IL-6, TNF-α, chemokines—CXCL10 (IP-10), CCL2 (MCP-1), and CCL3 (MIP-1α), is characterized by low IFN-I levels in the blood at an early stage and increased IFN-I levels at a late stage of COVID-19 [197]. It is important to remember that IFN- λ1, which is produced due to the activation of RIG-MDA-5, TLR3 receptors, and RIG-I has the most significant protective role in infection of respiratory epithelial cells with SARS-CoV-2 [223]. It is important to remember that IFN-λ1, which is produced due to the activation of RIG-I, MDA-5, and TLR3 receptors, has the most significant protective role at the beginning of the SARS-CoV-2 infection of respiratory epithelial cells [223]. When compared to patients with a milder form of COVID-19, IFN-I, and IFN-λ2 expression appears to be much higher (particularly in the lower respiratory tract) in patients with a severe course of the disease [223].

These findings back up the theory that IFNs have an ambiguous role in the airways. Effective initiation of IFN production in the upper respiratory tract can lead to faster virus clearance and possibly limit virus transmission to the lower respiratory tract. When the virus escapes immune control in the upper respiratory system, however, the generation of IFNs, which are considerably boosted in the lungs, is likely to contribute to the cytokine storm and tissue damage that occurs during COVID-19 progression to the critical stage. IFN produced the lowest (weakest) ISG response in the above example, but it significantly increased the surface expression of ACE2, which is crucial for SARS-CoV-2 penetration [224]. IFN-γ, secreted mainly by type 1 T-helper cells (Th1), is a key activator of type 1 proinflammatory macrophages (M1). The antiviral immune response of these cells is linked to the risk of phlogogenic substances damaging their tissues [4]. At the same time, IFN-I-III is primarily produced by virus-infected cells, whereas IFN- is primarily produced by lymphocytes activated by secondary immune factors but not infected by the virus. Therefore, the imbalance between the activation and limiting mechanisms of cellular stress in immunocytes induced by SARS-CoV-2 contributes to the disruption of both the innate and adaptive antiviral responses.

## 5. Dysfunction of the Adaptive Antiviral Response. The Alleged Role of the Autoimmune Process in the Pathogenesis of COVID-19

### 5.1. The Main Features of Immune Response Development and Its Dysfunctions in COVID-19

Possible variants of the strategy employed by viruses for evading immune response factors are screening for antigenic structures, induction of polyclonal lymphocyte activation, autoimmune response induction, blocking of the interaction between major histocompatibility complex (MHC) proteins on antigen-presenting cells (APC) and T-cell receptor (TCR), dysregulation of the cytokine network, an imbalance between the individual vectors of the immune response: i1 (Th1, macrophages–M1, normal killer cells–NK, cytotoxic T-lymphocytes–CTL), i2 (Th2, M2a, eosinophils, mast cells), i3 (Th17, M2b, neutrophils), i-reg (Treg, M2c) [225,226,227]. The implementation of these strategies can lead not only to an increase in the viral invasion but also to the development of secondary (bacterial and fungal) infections, local and systemic damage to tissues by variants of autoinflammatory and autoimmune reactions.

Five SARS-CoV-2 nsp (nsp7, nsp8, nsp9, nsp12, and nsp13) and structural proteins (S, E, M, and N) were found to include conserved peptides whose immunogenicity is determined by their ability to bind to MHC-I/II proteins [228].

RBD-specific antibodies identified in persons recovering from COVID-19 have been shown to protect against reinfection with SARS-CoV-2 [229]. Patients infected with SARS-CoV-2, on the other hand, show an early conversion of antitelogenesis from immunoglobulins of the IgM class to IgG and, to a lesser extent, IgA (7–14 days after the onset of symptoms) [225,229].

Anti-S-protein immunoglobulins produced for vaccines have also been shown to play a protective role in the prevention and reduction of COVID-19’s critical consequences [230,231]. The existence of IgG antibodies to S or N protein was related to a considerable reduction in the risk of SARS-CoV-2 re-infection in the next 6 months [232]. In general, only a few cases of reinfection have been reported over the world, implying long-term protective immunity [233]. In Qatar, only 243 people (0.18 percent) out of 133,266 laboratory-confirmed cases of SARS-CoV-2 had at least one subsequent positive result of detecting the RNA virus 45–129 days after the initial verification of COVID-19, and clinical evidence of reinfection was only established in 54 cases (22.2 percent) [234]. Meanwhile, several studies have shown that SARS-CoV-2 can survive for lengthy periods in the lack of antiviral IgG and that recovery from COVID-19 can occur in the absence of antiviral antibodies [235].

Antibody-dependent enhancement (ADE) is a phenomenon in which an attachment to antibodies does not destroy the virus, but rather facilitates its entry into the cell, promoting viral reproduction and virulence [225]. After attachment to the virus, antibodies against the S-protein mediate ADE via binding to FcR on different cells in the case of MERS-CoV and SARS-CoV-1 [236,237]. That said some researchers suggest that ADE can occur in SARS-CoV-2 infection [238,239,240].

Many SARS-CoV-2 proteins, including nsps, S, M, N, and orf3a, can serve as a source of Ag for antigen-specific CTL presentation (MHC-I + Ag) [241]. In addition, SARS-CoV-2 infection triggers Th1 development via involving MHC-II at the APC [242,243], which is accompanied by an accumulation of i1-response products in the blood, such as IFN-γ (primary producers–Th1), TNF-α, and neopterin (products M1) [244]. SARS-CoV-2 infects alveolar macrophages in the lungs, prompting them to produce chemoattractants for Th1. Th1 cells then release IFN-γ, which further activates resident and monocyte-recruited macrophages’ antiviral and proinflammatory activities [245]. As a result, a positive feedback loop develops, resulting in prolonged alveolar inflammation. Inadequately high M1 activation can cause changes in the COVID-19 inflammation zone in the lungs and intestines [246]. Excessive suppression of Th1 and IFN-γ production is related, on the other hand, to an inadequate rise in the i2-response in severe cases of COVID-19, and additional causes of IL-6 and IL-10 overproduction may be detected [247,248]. In such circumstances, low levels of IFN-γ in the blood are linked to the severity of the COVID-19 course [249].

Lymphopenia, which is related to decreased amounts of CD8+ T cells, as well as CD4+ Th and Treg cells in the blood, though to a lesser extent of decrease in B cells and NK cells, is another sign of immunological dysfunction in COVID-19 [225,250,251]. Furthermore, the severity of the patient’s condition is related to the degree of lymphopenia [250,252]. The number of neutrophils in COVID-19, on the other hand, frequently increases [253]. As the disease worsens, the neutrophil/lymphocyte ratio, which is characteristic of COVID-19, rises [254,255]. Many acute viral infections cause transient lymphopenia, but it usually resolves quickly [256,257]. Lymphopenia in COVID-19, on the other hand, can be more severe or long-lasting than in many other viral infections [248]. The following are the most likely causes of lymphopenia [251,258,259,260]:

- Migration of cells to the focus of inflammation;
- Direct infection of lymphocytes expressing ACE2 and other SARS-CoV-2 receptors, followed by their apoptosis or cytolysis;
- Apoptosis and pyroptosis of hyperactivated cells not infected with SARS-CoV-2;
- Metabolic and neuroendocrine changes caused by a viral infection lead to depletion and impairment of lymphoid cell regeneration.

Notwithstanding, the duration of lymphopenia in COVID-19 (up to several months after recovery) casts doubt on the essential role of lymphocyte migration to the inflammatory focus and infection of lymphocytes as the leading causes of lymphopenia.

A combined increase in the levels of IFN-γ and TNF-α plays a unique role in lymphocyte apoptosis in cytokine storm syndrome [261]. Both of these proinflammatory cytokines are linked to the immune response’s main antiviral vectors, i1 and TNF-α, as well as i3 [5]. High concentrations of these cytokines, on the other hand, can result in insufficient immune cell activation, dysfunction, and apoptosis. Patients with severe COVID-19 have a lower Treg/Th17 ratio, which is associated with high levels of proinflammatory cytokines and neutrophillia in the blood [262]. IL-17 and GM-CSF levels are elevated in patients with severe COVID-19 (i3 products). Furthermore, the recruitment of neutrophils, which is mediated by Th17 cells, has been shown to explain the destruction of lung tissue [263].

In severe cases, high values of cytokinemia in the form of a cytokine storm phenomenon are a sign of systemic inflammation, which is critical for the life of patients, and in some cases, MAS-like syndrome (macrophage activation syndrome) [4]. Furthermore, as previously mentioned, increased Th1 and M1 activation might harm tissues in the inflammatory foci. As a result, the evidence on IFN-’s pathogenetic role in COVID-19 is conflicting. Some publications point to IFN-’s protective role in antiviral protection as well as the efficacy of IFN- preparations in the treatment of this condition [264]. Other studies link elevated IFN levels in the blood with the severity of COVID-19 patients’ symptoms [265], as well as the ineffectiveness of IFN in antiviral protection of airway epithelial cells and its potential to induce ACE2 expression on intestinal epithelial cells [266]. The i1 factors promote the utilization of already infected epithelial cells but only weakly prevent their infection with SARS-CoV-2, which appears to be limiting the antiviral benefits of the i1 response in COVID-19.

The following are some of the mechanisms through which SARS-CoV-2 induces immunological dysfunction:

- Inhibition of MHC-I in infected cells, which makes it difficult for CTL to recognize them (orf8, Table 2);
- Screening of antigenic determinants of the S-protein by sugars to prevent the recognition of its antigenic sites by antibodies [75,76];
- Binding of orf8 to the IL-17 receptor and its activation on immunocompetent cells, which can contribute to immune imbalances [201];
- Possible APC dysfunctions associated with the action of the SARS-CoV-2 S-protein on PRR, SR, and other receptors on these cells (Table 1);
- Disturbance in immunocompetent cells of the balance between various signaling pathways and mechanisms of cellular stress by many factors of SARS-CoV-2 pathogenicity.

Antigen-specific T cells in COVID-19 were found to exhibit early differentiated effector memory phenotypes early in the recovery phase [267]. Patients with severe COVID-19 had a considerably higher frequency, breadth (diversity of subpopulations), and magnitude of memory T cell responses than patients with a moderate course of the disease, with the most prominent responses being elicited by proteins S, M, N, and orf3a [268,269]. Seventeen years after the 2003 SARS outbreak, people who have recovered from SARS (a disease caused by SARS-CoV-1) have long-lived memory T cells that respond to the SARS-CoV-1 N protein. These T cells showed persistent cross-reactivity with the N-protein SARS-CoV-2 [269]. However, the role of memory T cells in preventing SARS-CoV-2 invasion and COVID-19 complications is currently not fully understood.

Another problem in the development of an adaptive immune response in COVID-19 is the potential for autoimmune response development.

### 5.2. Possible Causes of the Autoimmune and Anti-Inflammatory Process in COVID-19

An autoinflammatory response resulting from antigen-specific activation of innate immunity components, or the action of autoantigen-specific T cells and antibodies, or a combined form [270] can cause damage to tissues. These common pathogenetic patterns of many infectious processes can manifest themselves in COVID-19 [243,271].

An autoimmune response directed against tissues is the sword of Damocles for highly organized species of vertebrates and humans [272]. Organisms with adaptive immunity have multi-stage barriers to the initiation and development of autoimmune response and an autoimmune process (autoimmune inflammation). However, all these barriers can be overcome in the presence of a genetic predisposition to the action of various triggering ontogenetic and environmental factors of autoimmunity [273,274,275].

The following factors play a crucial role in autoimmune aggression during infection [276,277,278]:

(1) Molecular mimicry of viral proteins.
(2) “Bystander activation”–the release of autoantigens from tissue damaged by viruses and inflammation.
(3) Violation of biological barriers in immunoprivileged organs (central nervous system, eye, testes, placenta), providing accessibility for adoptive immunity of potential autoantigens.
(4) Polyclonal activation of lymphocytes as a result of the action of superantigens (SAg) or for other reasons (potentially autoreactive clones of T and B lymphocytes can also be activated).
(5) “Epitope spreading”–when the targets of autoimmune responses do not remain fixed, but can be expanded by including other epitopes on the same protein or in other proteins in the same tissue.

It is important to remember that not every process of polyclonal lymphocyte activation leads to an autoimmune response, and not every autoimmune response results in tissue damage and the development of an autoimmune pro-inflammatory process, which does not always lead to the development of a formal (canonical) autoimmune disease [279].

Because of SARS-molecular CoV-2’s mimicry, the virus’s and humans’ proteins have analogous peptide sequences, and immune responses against SARS-CoV-2 are directed towards human proteins. As a result, the structural proteins S and E have several areas homologous to human proteins [280]. Therefore, the E-protein may play a role in the activation of an autoimmune response (after the viral particle has been degraded). Orf3a, orf7a, orf7b, orf8, and orf9b, non-structural proteins of SARS-CoV-2, may also be involved in this process [280]. In particular, molecular mimicry was revealed between the human SARS-CoV-2 and HSP proteins (60 and 90), which was linked to Guillain-Barré syndrome and other autoimmune illnesses [281]. It is worth noting that COVID-19’s immunological response (to HSP) can damage the vascular endothelial lining [282].

**Figure 5:**
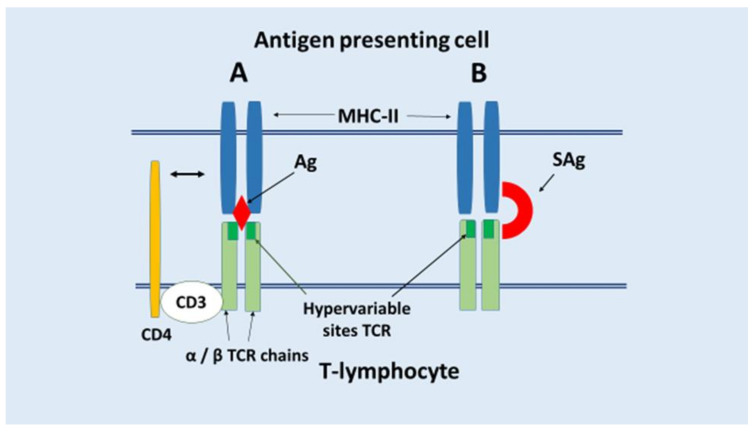
The induction of a clonal response of CD4+ T lymphocytes to particular antigenic peptides in association with MHC-II proteins to APC (**A**) and a polyclonal response (**B**) as a result of the action of superantigens. MHC-II proteins are displayed in blue, antigens (Ag, SAg) are shown in red, and chains of the T-cell receptor (TCR) are shown in light green, TCR antigen-specific (hypervariable) sites are indicated in dark green, and the CD4 coreceptor is shown in yellow. Arrows indicate antigen and receptor designations.

In COVID-19, it is currently hypothesized that superantigen-dependent (Sag) immune dysfunction pathways are also implicated in autoimmune and auto-inflammatory processes. Superantigens (Sags) are powerful polyclonal lymphocyte activators that are resistant to proteases and thermal denaturation [283,284]. The principal pathogenic activity of SAg is nonspecific binding to the β -chain of the TCR (less frequently to the α -chain) and simultaneously to? MHC-II (less frequently MHC-I) on the APC, resulting in polyclonal activation of T cells with TCR, including potentially autoreactive clones [285] (Figure 5).

Superantigens can activate up to 20–25 percent of T cells, compared to only 0.01% in the classical response, and at significantly greater Ag concentrations than SAg [284,286]. Most SAgs appear to have a common MHC-II binding site (called the generic site) that is unaffected by other Ags. Furthermore, each SAg has a preference for specific MHC alleles, which serve as possible genetic risk factors for autoimmune diseases [286]. SAg’s activating effect on T cells and B lymphocytes (expressing MHC-II), as well as innate immune cells, can eventually result in a cytokine storm and toxic shock [284,287].

Data have recently emerged relating the onset of multisystem inflammatory syndrome in children (MIS-C) to the probable impacts of SAg and the emergence of an autoinflammatory process in COVID-19. Moreover, MIS-C is a delayed and severe consequence of SARS-CoV-2 infection that affects previously healthy children [288,289]. It mimics toxic shock syndrome and certain symptoms of Kawasaki’s illness. Adults have been diagnosed with a comparable condition (multisystem inflammatory syndrome in adults, MIS-A) [290]. The MIS-C and MIS-A hypotheses [288,289,290,291] look at the pathophysiology of these disorders through the lens of autoinflammation and autoimmunity. In this scenario, the SAg-like motif is located close to the SARS-CoV-2 spike protein’s S1/S2 cleavage site [291]. The area containing this motif has a strong affinity for the V-domains of both the α- and β-chains of the TCR, and it shares structural similarities with Staphylococcal enterotoxin B (SEB), one of the most potent SAg [292,293]. SEB binds not only MHC-II and TCR proteins, but also contact interaction receptors CD28 (on T cells) and B7 (on APC), which play a key role in the activation of these cells, including the generation of proinflammatory cytokines [294].

The SARS-CoV-2 S-protein can form a ternary complex: S + TCR + MHC II, according to in silico computational models [295]. A rare mutation of the S protein (D839Y/N/E) from the European strain of SARS-CoV-2 [295] can enhance the virus’s interaction with T cells. The hyperinflammatory polyclonal proliferation of T-lymphocytes in MIS-C has also been linked to MHC-I, or more specifically, the MHC-I alleles A02, B35, and C04 [296]. Patients with MIS-C had a distorted TCR memory T cell repertoire, autoimmune IgG reactive to endothelium and other tissues, and hyperactivated cytotoxic CD8+ T cells and NK cells [297,298,299]. Overall, MIS-C is a serious but rare COVID-19 complication. Secondary hemophagocytic lymphohistiocytosis is another serious COVID-19 consequence linked to polyclonal activation of lymphocytes and aberrant activation of innate immune cells (HLH). It frequently presents as a MAS-like syndrome, which may be one of the mechanisms for the development of a cytokine storm and systemic inflammation, both of which are life-threatening [4]. It is worth remembering that systemic inflammation is a multifaceted, phase-specific process with hyperergic and depressed manifestations [272,300]. As a result, a cytokine storm, if we define it as critical cytokine levels, should have quantitative characteristics that greatly exceed reference values, such as TNF-α concentration in blood plasma > 200 pg/mL (at N 8 pg/mL) and IL-6 concentration in blood plasma > 1000 pg/mL (at N 5 pg/mL) [301]. Lower cytokine concentrations, in our opinion, necessitate the adoption of integral criteria for assessing the systemic inflammatory response and systemic inflammation. Currently, there are no quantitative verification criteria that distinguish cytokine storm syndrome from non-critical forms of hypercytokinemia [302]. This scenario appears to allow it to be mislabeled as a “cytokine storm,” which more closely resembles a “storm in a glass of water,” “storm in a teacup,” or “storm in a cup of water” COVID-19 [4].

Meanwhile, the processes of polyclonal lymphocyte activation and latent autoimmune response can affect a range of systems, including the immunological, neuroendocrine, cardiovascular, pulmonary, cutaneous, and digestive systems [286,303]. However, in the presence of additional risk factors, these same processes can result in the development of classical autoimmune diseases after COVID-19 [304,305,306]. Nevertheless, the chances of developing these diseases as a result of the COVID-19 transfer are modest. In COVID-19, nonclassical symptoms of an autoimmune response are more prevalent. Thus, SARS-CoV-2 protein mimicry of three human proteins, DAB1, AIFM, and SURF1, found in the pre-Bötzinger complex (preBötC) of the brain stem, may contribute to COVID-19 respiratory failure [307]. Other autoantibodies common in many autoimmune illnesses, such as antinuclear antibodies (ANAs), anticytoplasmic neutrophil antibodies (ANCA), and antiphospholipid antibodies (APL), were discovered in 45 percent of COVID-19 patients [308]. However, in most situations, the pathogenetic relevance of these autoimmune factors remains unclear.

The problem of “long COVID”, also known as “condition after COVID-19, unspecified” (International Classification of Diseases-10, ICD-10), “post-COVID-19 syndrome”, and “post-acute COVID,” may make the identification of hidden indications of an autoimmune response more important. Persistent fatigue, anhedonia, muscle weakness, sleep issues, anxiety or even depression, difficulty concentrating, myalgia and arthralgia, and autonomic dysfunction are all symptoms of long COVID [309,310,311,312,313]. In roughly 20% of patients, these symptoms persist for 3–6 months or longer after they have been cured of COVID-19. Long COVID can also be found in patients with mild COVID-19 [314]. As a result, following severe COVID-19 [311], long COVID requires special attention. The mechanisms of long COVID pathogenesis are yet unknown. Organ damage, post-viral syndrome, post-critical care syndrome, and other factors have been postulated as possible causes. Given the immunopathogenesis of COVID-19, its role in long-term immunological dysfunction, particularly the autoimmune response, appears to be a distinct possibility. In addition, new evidence suggests that SARS-CoV-2 can infect the central nervous system and the brain cells, particularly microvascular endothelial cells [315,316]. Hypoxia and cytokine storms, when combined with viral invasion, can compromise the blood-brain barrier (BBB) and cause brain tissue damage [306,307]. SARS-CoV-2 is thought to cause irreversible consequences in the brain due to BBB degradation, including dementia in predisposed people [315]. It is worth noting that BBB destruction plays a key role in the immunopathogenesis of autoimmune neurological disorders, as this vascular interface serves as an entryway for peripheral immune cells and effector molecules to reach an immunoprivileged target organ [317,318,319].

## 6. Conclusions

Despite the large-scale sanitary and preventive efforts taken over the last two years, the COVID-19 pandemic remains active, and optimistic forecasts about its end have yet to be justified. There is currently no scientifically validated forecast of the infection’s future behavior. SARS-CoV-2 can diminish its virulence with a relatively favorable variant, and its offspring strains or species will vanish or, like “colds”–CoV (HCoV-OC43 and HCoV-HKU1), induce episodes of common seasonal respiratory infections [320]. However, because of the virus’s unique biochemistry, less positive alternatives for adapting SARS-CoV-2 to human society are also possible.

SARS-CoV-2 has a high level of genetic variability, a large genome, and a large proteome, all of which are accompanied by pronounced polyfunctionality of its proteins. As a result, SARS-CoV-2 can infect target cells in the upper respiratory tract, epithelium of the alveoli (mainly type 2 alveocytes), and the intestines, after initial contact. SARS-CoV-2 also inhibits the early protective effects of type I and III IFNs and other innate antiviral defense systems. SARS-CoV-2 additionally causes the formation of symplasts of infected and healthy cells and extracellular vesicles containing viruses, which help it bypass the epithelium’s biological barriers and spread throughout the body. Since the SARS-CoV-2 S-protein can recognize not only the major receptor (ACE2) and its coreceptors, but also other (alternative) receptors, SARS-CoV-2 can potentially infect a wide variety of target cells in the host organism’s tissues.

Like many other acute viral infections, COVID-19 can, with a progressive course, implement four main stages of pathogenesis:

(1) Invasion and forced replication of the virus in integumentary tissues, primarily in the epithelium of the respiratory tract and intestines. The key mechanisms of this stage are the recognition of target cells by the virus through receptor structures and blockade of the primary (early) IFN-I-III response.
(2) Virus-induced loss of the barrier functions at the focus of inflammation through the dysregulation of the innate and then the adaptive immune response controlled by the pathogenicity factors. The selective suppression of specific antiviral mechanisms, the indiscriminate polyclonal activation of lymphocytes, the development of hypoinflammation according to the variant of an autoinflammatory process, and in some cases, a latent or even clinically manifest autoimmune process are all factors that assist viral expansion during this period.
(3) Generalization of the virus in the body, systemic changes in the immune status, and other parameters of homeostasis with a high risk of critical complications.
(4) The stage of long-term consequences of a viral infection associated with long-term persistence of the virus in the body, the consequences of primary (viral) and secondary (autogenous) tissue damage, including possible breach of the BBB integrity, endothelial dysfunction, hypercoagulation, and persistent changes in the immune status.

SARS-CoV-2 features a particularly high degree of genetic variability, owing to its large genome and proteome size, as well as its proteins’ pronounced polyfunctionality. The high variability of the spike protein, in particular, influences the possibility of invasion of many different cell types, including alveolocytes, not only via the main receptor (ACE2) but also via a variety of coreceptors and alternative receptors (Table 1). As a result, the effects of the SARS-CoV-2 S protein on immunocytes’ PRR (including TLR), SR, IL-17 receptor, and other sensory molecules can be dysfunctional to the immune system, resulting in tissue damage and viral replication in various cells.

The ability of the S-protein to induce the formation of cellular symplasts and to be screened by sugars, the potential for the development of the ADE phenomenon, and the use of extracellular vesicles by the virus may be its mechanisms of protection against the factors of the humoral immune response, including those associated with vaccines.

Almost all SARS-CoV-2 proteins (Table 2) are multifunctional pathogenic factors that act on various mechanisms of antiviral immunity. However, their most widespread effect is probably associated with the blockade of almost all stages of initiation and implementation of the IFN response (Figure 3 and Figure 4). These mechanisms allow the virus to effectively overcome the barrier functions of integumentary tissues in the initial period of the disease and contribute to the dysfunction of the immune response associated with IFN-γ at later stages of the infectious process. In addition, this circumstance reduces the efficacy of the use of IFN synthesis inducers as the COVID-19 therapy.

Another feature of the pathogenicity factors of SARS-CoV-2 is its ability to act on almost all the main processes of proinflammatory cellular stress development in various types of cells, including the formation of inflammasomes, oxidative stress, the response of noncoding RNAs, autophagy, expression of universal transcription factors of cellular stress (for example, NF-κB), and the formation of a secretory phenotype associated with cytokine networks. These mechanisms provide the virus with an additional opportunity to replicate in various cells, induce immune dysfunction and form abnormal inflammation patterns.

There is now no doubt that COVID-19 can develop an auto-inflammatory and autoimmune process, which may well lead to complications such as MAS-like syndrome and MIS-C/MIS-A. These complications, along with other causes, contribute to systemic microcirculatory disorders and other life-critical manifestations of systemic inflammation. Therefore, all of the main factors for the emergence of an auto-immune response can potentially manifest themselves in COVID-19, including molecular mimicry of many SARS-CoV-2 proteins, “Bystander activation”, “Epitope spreading” (in conditions of tissue destruction), breach of the biological barriers in immunoprivileged organs, polyclonal activation of lymphocytes, and pathological activation of APC (as a result of the action of SAg).

Currently, a comprehensive assessment of these mechanisms is one of the most relevant scientific directions for studying the pathogenesis of not only COVID-19 but also other viral infections [320,321,322,323,324].

A special problem of the infection under consideration is the “long COVID” phenomenon, primarily associated with long-term disorders of the central nervous system, persistent changes in the immune system (including long-term lymphopenia), and complications in other organs. Probably, we will see a more complete picture of the pathogenesis of “long COVID” as a result of further research, including clarification of the role of autoimmune and autoinflammatory processes, impaired BBB, viral persistence, and comorbid vascular pathologies.

The warning signs of COVID-19 are:

- The presence, in some cases, of long-term persistence of SARS-CoV-2 in the body in immunodeficient patients [324];
- The presence, in about every fifth case, of long-term consequences caused by the transferred infection in the form of a long COVID;
- Longer (in comparison with SARS-CoV-1 and MERS-CoV) retention of SARS-CoV-2 RNA in biological fluids in COVID-19 patients (up to 1–2 months) [325], as well as the detection of SARS-CoV-2 RNA in more than one out of ten patients with relapse within 60 days after recovery from primary infection [326];
- The presence of the COVID-19 clinic in some cases of relapse and the occurrence of infection in vaccinated patients who initially did not have clinical signs of immunodeficiency states [327,328];
- The development of a latent and, in some cases, clinically significant auto-immune response in COVID-19;
- The potential ability of the virus to actively overcome the factors of not only innate but also adaptive immune response.

**Figure 6:**
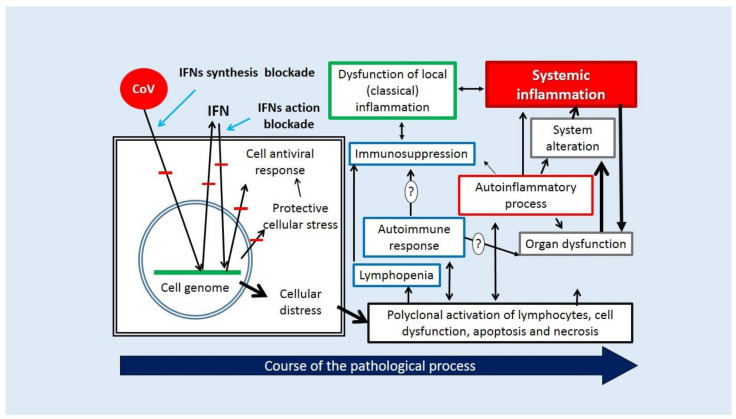
COVID-19 pathogenesis (pathokinesis) stages. (1) Infection of integumentary tissue cells, overriding of IFN-dependent systems of innate immunity cellular defense, blockade or hyperstimulation of cellular stress signaling pathways, and other consequences of cellular distress. (2) Dysfunction of adaptive immunity and canonical inflammation processes in the zone of invasion, breach of the focus of inflammation’s barrier function, dysfunction of the damaged organ, and virus generalization in the body. (3) Increasing changes in the body’s homeostasis, formation of the systemic alteration phenomena, and development of systemic inflammation. IFN induction routes and IFN action in infected cells are indicated by blue arrows. Other arrows indicate the directions in which distinct pathogenetic processes interact with one another. Blocks of IFN generation and their impact on cells that generate SARS-CoV-2 proteins are shown in red lines. Processes associated with immune dysfunction are highlighted in blue boxes; autoinflammatory processes are highlighted in red; the focus of inflammation dysfunction is highlighted in green boxes; tissue dysfunction is highlighted in black boxes, and systemic inflammation is highlighted in red fill boxes.

Thus, depending on the characteristics of a particular strain of SARS-CoV-2, environmental factors, the presence of comorbid pathologies, and the state of the immune system of the body, the dynamics of COVID-19 may stop at its initial stages (including subclinical variants) or reach the terminal, critical life stages of the disease associated with multiple systemic disorders of homeostasis (Figure 6).

## Figures and Tables

**Table 1:** Prospective alternative and cofactorial receptors for ACE2 SARS-CoV-2.

Receptor [Ref]	Expression on Cells	Receptor Functions
ACE2 [83,84]	Epithelial cells, macrophages, platelets, endothelial cells, smooth muscle cells, many other cells.	Main receptor for SARS-CoV-1 and SARS-CoV-2; neutralization of Ang II; formation of anti-inflammatory 1-7 Ang.
Chondroitin sulfate [55,79]	Most of the cells.	Component of the cell glycocalyx; coreceptor for SARS-CoV-2 and several other viruses.
Neuropilin 1 (NRP1, CD304) [85,86,87]	Nerve cells of the brain and nasal cavity, endothelial cells.	Coreceptor for binding SARS-CoV-2; coreceptor for many growth factors. The receptor function of NRP-1 depends on the cleavage of S-protein by furin.
AXL [88]	Expression of AXL > ACE2 in many tissues, and in the lungs and bronchi.	A putative alternative receptor for SARS-CoV-2; binds several growth factors and phosphatidylserine.
CD147 (Basigin) [89,90]	It is widely expressed in human tissues, highly expressed on cells of the immune system.	The coreceptor and activator of ACE2 expression; receptor for many other viruses; polyfunctional membrane chaperone that binds and is activated by cyclophelin A and B.
GRP78 (BiP, HSPA5) [91,92,93,94,95]	On different cells.	Coreceptor for SARS-CoV-2; an inducible HSP of the HSP70 family; a cellular stress factor; a gateway for many viruses.
SR-B1 [35]	Proliferating cells, hepatocytes, macrophages, adrenal cells.	Coreceptor for SARS-CoV-2; the main receptor for the hepatitis C virus, the HDL receptor; SR.
SR-H2 (Stabilin-2) [96]	Macrophages, endothelial cells.	SR; a lectin; could potentially be involved in the uptake of SARS-CoV-2 by macrophages.
CD209L (L-SIGN) [97,98,99]	Endothelial cells, alveolar epithelium.	An independent receptor and a cofactor for ACE2 in SARS-CoV-2 infection; cell adhesion factor.
CD209 (DC-SIGN) [97,98,100]	Macrophages (including alveolar), dendritic cells.	SR and alternative receptor for SARS-CoV-2; Binding of various PAMPs, including SARS-CoV-1/2 and other viruses, cell adhesion.
SR-E2 (Dectin-1, CD369) [100]	Macrophages, monocytes, dendritic cells, neutrophils, eosinophils.	SR, binds PAMP (β-1,3 and β-1,6 glycans). Can recognize glycans on the SARS-CoV-2 S protein and integrate with TLR2 and TLR4.
SR-E3 (CD206, Mannose receptor 1) [100]	Macrophages, monocytes, dendritic cells.	SR, can bind mannose-rich microbial glycans, SARS-CoV-2 S1 protein and integrate with TLR2 and TLR4.
DPP4, (CD26) [101,102]	It is widely expressed on the surface of many cell types, including the respiratory epithelium.	The main receptor for MERS-CoV, a possible alternative receptor for SARS-CoV-2; a multifunctional receptor.
ANPE [63,103].	Various epithelial cells, neutrophils.	A putative alternative receptor and ACE2 coreceptor for SARS-CoV-2; a well-known receptor for the entry of many CoVs.
ENPEP (CD249) [63,104].	The expression pattern is similar to that of ACE2.	A putative alternative receptor and ACE2 coreceptor for SARS-CoV-2. Participates in the regulation of vascular tone.
ASGr1 [105]	Hepatocytes.	Receptor for many viruses, including hepatitis B virus; a possible alternative receptor for SARS-CoV-2.
KIM-1/TIM-1 [106]	Epithelium of the lungs and kidneys.	Probably an alternative ACE2 receptor for SARS-CoV-2.

Ang—angiotensin; ANPE—alanylaminopeptidase; AXL—tyrosine-protein kinase receptor UFO; DPP4—dipeptidyl peptidase 4; ASGr—asialoglycoprotein receptor 1; ENPEP—CD249, Glutamylaminopeptidase; HDL—high-density lipoprotein; HSP—heat shock protein; KIM-1/TIM-1—kidney injury molecule-1/T cell immunoglobulin mucin domain 1; PAMP—pathogen-associated molecular patterns; SR-E3—CD206, Mannose receptor 1; SR—scavenger receptors; ACE2—angiotensin-converting enzyme 2.

**Table 2:** Functions of invasiveness and virulence of individual proteins of SARS-CoV-2.

Proteins	Functions
Nsp1	Suppresses the host protein synthesis (including IFN-I and RIG-I) through association with ribosomes [155,156]. Inhibits IRF3 phosphorylation and IFN production; suppresses ISG induction due to Tyk2 and STAT2 depletion and inhibition of STAT1 phosphorylation [40,157]. Changes the structure and function of the cytoskeleton [158].
Nsp2	Can participate in the binding of nucleic acids and the regulation of intracellular signaling pathways [159]. Participates in the processes of viral replication [160]. Proteins that interact with nsp2 are involved in several biological processes, such as endosomal transport and translation [161]. Changes the synthesis and modification of lipids [158].
Nsp3	Papain-like protease (PLpro) and deubiquitinase. Participates in the proteolysis of 1a/1ab polyproteins [10,162]. Cleaves IRF3 [163]. By removing ubiquitin in the structures of signaling pathways, they disrupt the development of antiviral cellular stress, including translocation of IRF3 into the cell nucleus and inhibition of RIG-I, STING, TRAF3, TBK1, IRF3 [164]. Nsp3 SARS-CoV-1 c promotes inactivation of conjugated ubiquitin-like molecules such as interferon, which is involved in different pathways of the innate antiviral immune response regulation [165,166]. However, the deubiquitinase and IFN-blocking functions of nsp3 SARS-CoV-2 seem to be significantly lower than that of nsp3 SARS-CoV-1 [150]. Participates in the RTC formation [167].
Nsp4	An ER-localized transmembrane protein (as nsp3 and nsp6), is considered to be involved in the assembly of virus-induced cytoplasmic double-membrane vesicles [168]. Participates in the RTC formation [167].
Nsp5	3-chymotrypsin-like “main” protease (3CLpro) is involved in the proteolysis of 1a/1ab polyproteins [10]. Inhibits the translocation of IRF3 into the cell nucleus and promotes the degradation of IRF3 [169]. It regulates (by proteolysis) the development of pro-inflammatory stress in different directions: activating the formation of NLRP12 by inflammasomes and inhibiting kinase - TGF-beta activated kinase 1 (MAP3K7 - mitogen-activated protein kinase 7) binding protein 1 (TAB1) [163]. Participates in the RTC formation [167].
Nsp6	Inhibits the phosphorylation of IRF3, STAT1, and STAT2 [110]. Potentially contributes to SARS-CoV-2 infection by impairing the ability of autophagosomes to deliver viral components to lysosomes for degradation [170,171]. Probably, along with orf7a, can interact with sigma receptors, which are involved in lipid remodeling and the ER stress response [161]. Participates in the RTC formation [167].
Nsp7	During viral RNA replication, the nsp12 cofactor forms a primase complex with nsp8 [167,172]. Participates in the RTC formation [167,173].
Nsp8	The nsp12 cofactor in viral RNA replication [162]. Participates in the formation of the RTC [167,173]. Forms a primase complex with nsp7, which plays a decisive role in the regulation of the activity of RNA-dependent RNA polymerase (RdRP) nsp12. [174,175]. Suppresses the transport of membrane proteins in cells infected with SARS-CoV-2, disrupts IFN secretion [176].
Nsp9	RNA-binding protein, interacting with nsp12, is one of the key factors of RTC [173,175]. Suppresses the transport of membrane proteins in cells infected with SARS-CoV-2, disrupts the secretion of IFN and some other cytokines [176]. Interferes with the activation of IRF3 and the induction of IFN synthesis by inhibiting TBK1 [20,138].
Nsp10	The cofactor of nsp14 and nsp16 forms functional complexes with them upon methylation of viral RNA [154,172]. Acting on NKRF (NF-κB-repressive factor), can regulate the levels of IL-8, NKRF and form a unique immune signature in COVID-19 [177]. Participates in the RTC formation [167].
Nsp11	Includes only 13 amino acid residues [178]. The role of the nsp11 protein in cells infected with CoV has not yet been studied [179].
Nsp12	RNA-dependent RNA polymerase is a key enzyme mediating the synthesis of all viral RNA molecules [162]. A key component of RTC [167,173,175].
Nsp13	Helicase, 5′-triphosphatase. Inhibits the phosphorylation of TBK1, which leads to a decrease in IRF3 activation, inhibits the phosphorylation of STAT1 and STAT2 [40]. Participates in viral replication [170]. Changes the structure and function of the cytoskeleton [158]. Participates in the RTC formation [167,173,175].
Nsp14	3′-5 ′exoribonuclease and N-7-methyltransferase. Inhibits nuclear translocation of IRF3 [150]. Participates in the RTC formation [167,175]. As an exonuclease, it provides the ability to correct errors in the RNA synthesis complex, which allows SARS-CoV-2 to maintain its large genome [180].
Nsp15	NendoU, a uridylate-specific endoribonuclease. Inhibits nuclear translocation of IRF3 [150]. Participates in the RTC formation [167,175]. Meanwhile, nsp15-deficient CoVs are viable and can replicate [181], but with some delay and errors [182]. Another possible mechanism of nsp15 action is an evasion of viral double-stranded RNA recognition by host sensors in macrophages, including MDA5, PKR, and OAS/RNaseL [182].
Nsp16	2-Oʹ-methyltransferase, which blocks the recognition of viral RNA by PRR [183,184] by forming the morpho-functional complexes with nsp10 [174]. Participates in the RTC formation [167,175].
Orf3a	Disrupts the IFN signaling pathways by inhibiting STAT1 phosphorylation [40,177]. Can regulate the effects of IL-6 action by inhibiting STAT3 and STAT5 [177]. Promotes lysosomal degradation of the α-chain of the IFN-I receptor (IFNAR1) and induces ER stress (shown in SARS-CoV-1) [185,186]. Forms transmembrane ionic potassium-specific channels that facilitate the release of the virus from the cell [187]. It is assumed that viral ion channels can also promote membrane fusion and regulate viral replication and/or the packaging of genomic RNA into viral particles [187]. Activates NF-kB, NLRP3 inflammasomes, promoting cytokine storm [188,189]. Blocks the fusion of autophagosomes with lysosomes, contributing to the survival of the virus [136].
Orf3b	Interferes with nuclear translocation of IRF3 [190]. Can cause a specific antibody response as N and orf8 proteins [191]. Orf3b is not expressed in many strains of SARS-CoV-2 [150], however, SARS-CoV-2 orf3b can suppress IFN-I induction more efficiently than its orthologue SARS-CoV-1 [190].
Orf6	Inhibits IRF3 (via action on TBK1) phosphorylation [177]. Binds to KPNA2 (karyopherin subunit alpha 2) and the Nup98-Rae1 complex, which inhibits nuclear translocation of IRF3 and STAT1 [40,150,192]. Causes changes in signaling pathways that include the activation of apoptosis through caspase-3-mediated, ER stress, and JNK-dependent pathways (JNK-c-Jun N-terminal kinases) [158]. Inhibition of STAT1 causes compensatory hyperactivation of STAT3 in cells infected with SARS-CoV-2, which promotes overproduction and activation of plasminogen activator inhibitor-1 (PAI-1) and can lead to coagulopathy [193].
Orf7a	Inhibits the STAT2 phosphorylation [40]. According to the results of in silico calculations, can interact with sigma receptors, as nsp6 [194]. Has structural homology to ICAM-1, binds to Mac-1 and the integrin receptor LFA-1 on leukocytes. The orf7a expression leads to apoptosis, blocking the cell cycle, activation of NF-kB, and mitogen-activated protein kinase (MAPK) [195]. Binds to CD14 (TLR4 cofactor), promoting the production of proinflammatory cytokines [196]. Promotes the release of SARS-CoV-2 from the cell membrane by inhibiting the membrane protein BST-2 [197].
Orf7b	Inhibits STAT1 and STAT2 phosphorylation [40].
Orf8	Interacts with a variety of host proteins and blocks the class 1 major histocompatibility complex (MHC-I) protein in the ER lumen [198,199]. Inhibits IFN signaling pathways and activation of ISG promoters [200]. Orf8, being secreted into the extracellular space can activate the IL-17 signaling pathway by interacting with the host’s IL17RA [201]. Forms cation channels upon assembly in the lipid bilayer, like orf3a and E-protein [202].
Orf9b	Blocks the signaling pathways from TNF receptors by acting on TRAF3 and TRAF6 (TNF receptor-associated factor 3 and 6), disrupts IFN-I synthesis, and induces ATG5-mediated autophagy in host cells [189]. Blocks TOM70, a key adapter that transmits an antiviral signal from the mitochondrial RLR/MAVS pathway to TBK1/IRF3 to induce an IFN response [203,204]. Interacts with RIG-I, MDA-5, MAVS, STING, and TBK1, prevents phosphorylation and nuclear translocation of IRF3, NF-κB activation, and inhibits TRIF (TLR adapter) [205,206].
S	Plays a key role in the process of receptor recognition and cell membrane fusion [207]. The structure of this protein allows it to interact with many receptors in host cells (Table 1).
E	Promotes the assembly and release of the virus, has the properties of a viroporin membrane channel, which can contribute to damage to the epithelial barrier, the pathogenesis, and the severity of COVID-19 [208]. The function of the ion channel is associated with the activation of inflammasomes and the development of hyperinflammation in the lungs [209,210]. These effects of E-protein are capable of initiating a cytokine storm and the development of an experimental analog of ARDS [211].
M	The dominant structural protein can bind to other structural proteins such as S and E and determines the shape of the viral envelope [210]. Participates in the packaging of the genome in the viral particle, structures the viral particle [172]. Inhibits STAT1 phosphorylation [40]. M interacts with RIG-I, MAVS, and TBK1, thus preventing the formation of a multiprotein complex containing RIG-I, MAVS, TRAF3, and TBK1, and subsequently preventing phosphorylation, nuclear translocation, and activation of IRF3 [212].
N	Binds viral RNA and protects the viral genome, participates in the assembly of the genomic RNA of the virus [172]. Modifies the signaling pathway of the cytokine TGF-β, blocking the apoptosis of infected host cells, but can also induce apoptosis by activating the mitochondrial pathway [158]. Promotes hyperinflammation by activating NLRP3 inflammasome [213]. It interferes with RIG-I activation, interferes with the association between TBK1 and IRF3, and prevents nuclear translocation of IRF3 [214]. Inhibits the formation of G3BP1-dependent stress granules during the development of the cell’s antiviral response and blocks the host mRNA [215].

RTC—replication/transcription complex includes: nsp3, nsp4 and nsp6, which are involved in the formation of viral RNA synthesis sites, basic protease (nsp5), nsp nsp8 primase complex, RNA-binding protein nsp9, basic RNA-dependent RNA polymerase (nsp12), helicase/triphosphatase (nsp13), exoribonuclease (nsp14), endonuclease (nsp15), and N7- and 2’O-methyltransferase (nsp10/nsp16), ER—endoplasmic reticulum.
